# Load-independent effects of empagliflozin contribute to improved cardiac function in experimental heart failure with reduced ejection fraction

**DOI:** 10.1186/s12933-020-0994-y

**Published:** 2020-02-08

**Authors:** Kim A. Connelly, Yanling Zhang, Jean-François Desjardins, Linda Nghiem, Aylin Visram, Sri N. Batchu, Verra G. Yerra, Golam Kabir, Kerri Thai, Andrew Advani, Richard E. Gilbert

**Affiliations:** Keenan Research Centre, Li Ka Shing Knowledge Institute, St. Michael’s Hospital, 61 Queen Street East, Toronto, M5C 2T2 ON Canada

**Keywords:** Sodium–glucose linked co-transporter 2 inhibitor, Heart failure reduced ejection fraction, Systole, Diastole

## Abstract

**Background and aims:**

Sodium–glucose linked cotransporter-2 (SGLT2) inhibitors reduce the likelihood of hospitalization for heart failure and cardiovascular death in both diabetic and non-diabetic individuals with reduced ejection fraction heart failure. Because SGLT2 inhibitors lead to volume contraction with reductions in both preload and afterload, these load-dependent factors are thought to be major contributors to the cardioprotective effects of the drug class. Beyond these effects, we hypothesized that SGLT2 inhibitors may also improve intrinsic cardiac function, independent of loading conditions.

**Methods:**

Pressure–volume (P–V) relationship analysis was used to elucidate changes in intrinsic cardiac function, independent of alterations in loading conditions in animals with experimental myocardial infarction, a well-established model of HFrEF. Ten-week old, non-diabetic Fischer F344 rats underwent ligation of the left anterior descending (LAD) coronary artery to induce myocardial infarction (MI) of the left ventricle (LV). Following confirmation of infarct size with echocardiography 1-week post MI, animals were randomized to receive vehicle, or the SGLT2 inhibitor, empagliflozin. Cardiac function was assessed by conductance catheterization just prior to termination 6 weeks later.

**Results:**

The circumferential extent of MI in animals that were subsequently randomized to vehicle or empagliflozin groups was similar. Empagliflozin did not affect fractional shortening (FS) as assessed by echocardiography. In contrast, load-insensitive measures of cardiac function were substantially improved with empagliflozin. Load-independent measures of cardiac contractility, preload recruitable stroke work (PRSW) and end-systolic pressure volume relationship (ESPVR) were higher in rats that had received empagliflozin. Consistent with enhanced cardiac performance in the heart failure setting, systolic blood pressure (SBP) was higher in rats that had received empagliflozin despite its diuretic effects. A trend to improved diastolic function, as evidenced by reduction in left ventricular end-diastolic pressure (LVEDP) was also seen with empagliflozin. MI animals treated with vehicle demonstrated myocyte hypertrophy, interstitial fibrosis and evidence for changes in key calcium handling proteins (all p < 0.05) that were not affected by empagliflozin therapy.

**Conclusion:**

Empagliflozin therapy improves cardiac function independent of loading conditions. These findings suggest that its salutary effects are, at least in part, due to actions beyond a direct effect of reduced preload and afterload.

## Background

Loss of myocardial mass due to myocardial infarction remains the major cause of heart failure with reduced ejection fraction (HFrEF) [1]. The loss of contractile function leads to neurohumoral activation with expansion of extracellular fluid volume that not only precipitates the congestive manifestations of heart failure such as dyspnoea and edema, but also leads to adverse cardiac remodelling with fibrosis and hypertrophy that further exacerbate the extent of cardiac dysfunction.

Reduction in hospitalization for heart failure (HHF) was a major finding in three large cardiovascular outcome trials, EMPA-REG Outcome, CANVAS and DECLARE TIMI 18, that examined safety and efficacy of the SGLT2 inhibitors, empagliflozin, canagliflozin and dapagliflozin respectively [2–4]. These findings suggest that SGLT2 inhibitors prevent heart failure. More recently, the DAPA HF trial demonstrated a reduction in HHF and CV death irrespective of glycemic status, demonstrating that not only does this class of agents *prevent* HHF, but these agents may be used as a bona fide treatment *for* HF [5]. The mechanisms that underlie this beneficial effect are, however, uncertain with hypotheses that fall broadly in two categories. The first category theorizes that given their ability to induce an osmotic diuresis, load-dependent mechanisms such as reductions in preload and afterload are the major contributors to reduced HHF with SGLT2 inhibitors. Others have suggested that load-independent mechanisms such as increased oxygen delivery as a consequence of increased hemoglobin or improved energetics from using the ketone bodies as fuel are equally, if not more, important.

In contrast to load-sensitive measures of cardiac function such as echocardiography, pressure–volume relationship analysis using conductance catheterization enables load-independent mechanisms and their contribution to changes in cardiac function to be assessed [6]. The requirement for cardiac catheterization, however, largely precludes such studies being done in the human setting. Accordingly, we sought to examine the effects of the SGLT2 inhibitor, empagliflozin, focusing on the load-insensitive measures of systolic function, preload recruitable stroke work relationship (PRSW) [7] and end-systolic pressure–volume relationship (ESPVR) [8] in a rodent model of experimental HFrEF.

## Methods

### Animals

10 week old Fischer F344 rats were randomized to undergo sham surgery or ligation of the left anterior descending (LAD) coronary artery to induce myocardial infarction (MI) of the left ventricle (LV), as previously described [9]. Following confirmation of infarct size with echocardiography 1-week post MI, animals were then further randomized to receive vehicle, or the SGLT2 inhibitor, empagliflozin (20 mg/kg/day by gavage), for 6 weeks.

Just prior to termination, animals underwent echocardiography and cardiac catheterization as described below. Following these procedures, animals were terminated and their hearts were harvested for structural and molecular measurements. Tibial length was measured to provide a morphometric index for cardiac hypertrophy and lung weight [10]. All animals were housed 2/cage at the St. Michael’s Hospital Animal Research Vivarium in a temperature-controlled (22 °C) room with a 12-h light/dark cycle and ad libitum access to commercial standard rat chow.

All animal studies were approved by the St Michael’s Hospital Animal Care Committee in accordance with the Guide for the Care and Use of Laboratory Animals (NIH Publication No. 85-23, revised 1996).

### Echocardiography

Transthoracic echocardiography was performed, as previously described [9], under light anaesthesia (1% isoflurane supplemented with 100% O_2_), prior to sacrifice. Images were acquired using a high-frequency ultrasound system (Vevo 2100, MS-250 transducer, Visualsonics, Toronto, ON). Two dimensional long-axis images of the LV in parasternal long- and short-axis views with M-mode measurements at mid-papillary muscle level and linear dimensions were analyzed offline (Vevo 2100 software v. 1.8) using the standard leading edge-to-leading edge technique by a single investigator, masked to treatment. Fractional shortening (FS%) was calculated according to the formula: FS% = (LVIDd − LVIDs)/LVIDd × 100, where LVIDd and LVIDs are end-diastolic diameter and end-systolic diameter respectively, as previously described. Three consecutive cardiac cycles were averaged for all analyses.

### Cardiac catheterization

Cardiac catheterization was performed as previously published [10]. Briefly, rats were anaesthetized with 2% isoflurane, intubated using a 14 gauge catheter and ventilated using a pressure controlled ventilator (TOPO ventilator, Kent Scientific, Torrington, CT). Adequacy of anesthesia was assessed by lack of response to surgical manipulation and loss of muscular tone. Rats were placed in the supine position on a water circulating heating pad and a 1.4F pressure–volume (PV) catheter (SPR-838; Millar Instruments, Inc., Houston, TX) was inserted into the right carotid and advanced into the left ventricle and PV loops were generated. All pressure–volume (PV) loops were obtained with the ventilator turned off for 5–10 s and the animal apneic.

Data were acquired and recorded with a MPVS ultra^®^ data acquisition system (Millar Instruments) and LabChart Pro software (CHART 8.1 ADInstruments Inc., Colorado Springs, CO) under steady-state and following inferior vena cava occlusion (preload reduction). Conductance signals acquired with the Millar catheter were calibrated with the estimated LV volumes derived from echocardiography by using a two-point calibration method, matching LV maximal and minimal conductance signals and end-diastolic and end-systolic volumes (EDV and ESV) measured in long-axis view. Using the pressure conductance data, functional parameters were then calculated, as previously reported [7].

### Histopathology

The extent of cardiac myocyte hypertrophy was determined on haematoxylin and eosin stained sections, as previously reported [11]. In brief, stained sections were scanned digitally by high resolution microscopy (Ultra-Resolution Digital Scanning System, Aperio Technologies Inc., Vista, CA), and images analyzed with The NDP view2 software (Hamamatsu Photonics, Hamamatsu City, Shizuoka Pref., Japan). Cardiac myocytes with elliptical nuclei in transverse section were selected. Diameter measurements were taken membrane to membrane across the narrowest point that crosses the nucleus. The average diameter of 30–50 myocytes per animal was measured, as previously described [12].

Changes in cardiac structure were assessed in a masked protocol in animals from each group. Sections were stained with fibrillar collagen subtypes I and III using specific antibodies (anti-type I collagen: Southern Biotechnology Associates, Inc. Birmingham, AL; anti-type III collagen: Biogenex, San Ramon, CA), and quantified as previously reported [7].

### Immunoblotting

Immunoblotting of the non-infarcted region of heart homogenates was performed with antibodies in the following concentrations: phosphorylated phospholamban (phospho-PLN) (Ser16) 1:1000 (A285); phospho-PLN (Thr17) 1:1000 (#sc-17024, Santa Cruz Biotechnology, Dallas, TX); phosphorylated Akt (Ser473) (#9271, Cell Signaling Technology, Danvers, MA); Ca^2+^/calmodulin-dependent protein kinase II (CaMKII) 1:1000 (#sc-5306, Santa Cruz Biotechnology); peroxisome proliferator-activated receptor gamma coactivator-1α (PGC-1α) 1:1000 (#ab54481, Abcam Cambridge, MA); SERCA2a 1:1000 (IID8F6); GAPDH 1:5000 (#2118 s, Cell Signaling Technology) [11]. Densitometry was performed using Image J version 1.39 (National Institutes of Health, Bethesda, MD). Values are shown as the fold change compared to the ratio of the protein of interest relative to GAPDH in the hearts of sham rats treated with vehicle.

### Statistics

Data are expressed as mean ± SEM unless otherwise specified. Between group differences were analyzed by two way ANOVA with Fisher’s least significance difference post hoc test. All statistics were performed using GraphPad Prism 6 for Mac OS X (GraphPad Software Inc., San Diego, CA). A p value of < 0.05 was regarded as statistically significant.

## Results

### Animal characteristics

Body weight was reduced in sham animals that had received empagliflozin, and while body weight was lower in animals with experimental MI, it was lower still in those that had received empagliflozin. Heart weight, indexed to tibial length (HW/TL), was elevated in animals with experimental MI though to a lesser extent in post-MI rats that had received empagliflozin when compared with untreated post-MI animals (Table 1).

**Table 1:** Animal characteristics

	Sham + control	Sham + empa	MI + vehicle	MI + empa
Body weight (g)	288 ± 4	267 ± 2*	271 ± 3*	257 ± 2*^,†^
Haemoglobin (g/L)	131 ± 7	144 ± 3*	143 ± 2*	150 ± 3^†^
Plasma sodium (mmol/L)	126 ± 2	128 ± 3	131 ± 1*	133 ± 1^†^
HbA1c (%)	4.8 ± 0.2	5.0 ± 0.1	4.7 ± 0.1	4.9 ± 0.1
HW indexed to tibial length (mg/mm)	18.1 ± 0.4	17.2 ± 0.3	18.9 ± 0.6	16.7 ± 0.3^†^
Lung weight indexed to tibial length (mg/mm)	25 ± 1	25 ± 1	31 ± 3	24 ± 1^†^

Data are expressed as mean ± SEM

N = 13–15 in sham Control and sham + Empagliflozin groups; N = 20 and 20 in MI Control and MI + Empagliflozin groups, respectively

*Empa* empagliflozin, *LV weight/TL* Left ventricular weight/tibial length, *LW/TL* lung weight/tibial length ratio

* p < 0.05 vs. sham control group

^†^p < 0.05 vs. MI control group

Lung weight, also indexed to tibial length (LW/TL), that provides an index of pulmonary congestion was numerically increased in animals with experimental MI and significantly reduced in those that had received empagliflozin (Table 1).

### Laboratory characteristics

In these non-diabetic animals, empagliflozin had no effect on glycaemia, as assessed by HbA_1c_, in both sham and post-MI groups. Hemoglobin was higher in rats that had received empagliflozin in both sham and post-MI groups consistent with plasma volume contraction and/or increased erythropoiesis (Table 1).

### Echocardiography

**Fig. 1:**
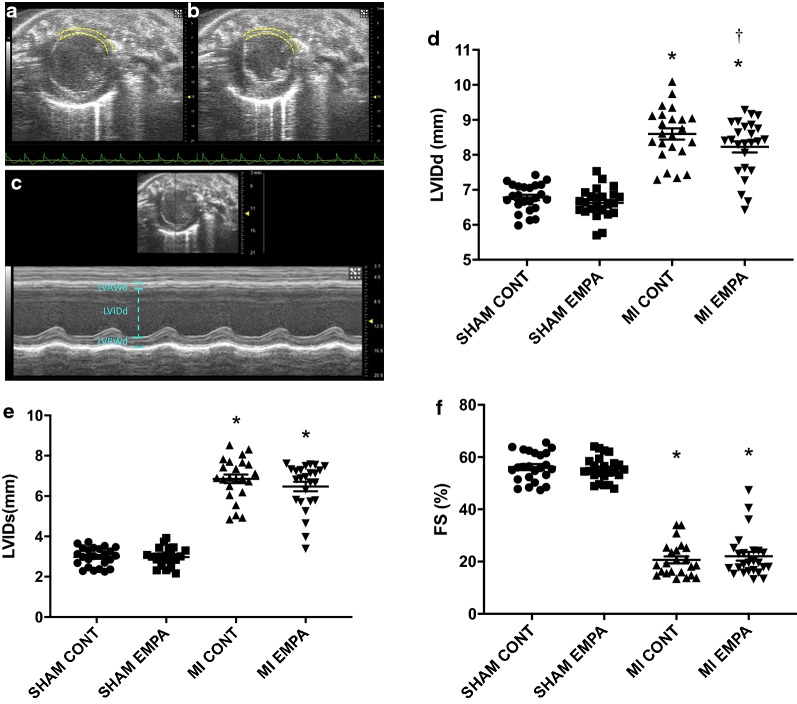
Echocardiographic parameters: Representative parasternal short axis views of the LV (Bmode) in diastole (**a**) and systole (**b**) 1-week post LAD ligation. The anterolateral infarct is outlined with dotted yellow lines. M-mode imaging (**c**) reveals thinning of the left ventricular anterior wall (LVAWd), increased internal diameter (LVIDd) and reduced function. **d**–**f** represent quantitation of LV internal diameter in diastole, LV internal diameter in systole and fractional shortening. MI reduced FS and led to ventricular dilatation. LVIDd was reduced by empagliflozin therapy. *p < 0.05 MI versus sham, ^†^p < 0.05 versus MI control

Myocardial infarction was confirmed in all randomised animals 1 week post LAD ligation, with an average circumferential extent of MI similar prior to randomization to either treated and untreated groups (32 ± 6% MI + vehicle and 32 ± 5% MI + EMPA, p = NS). Fractional shortening was impaired in animals with experimental MI when compared with sham rats but was unaffected by empagliflozin (Table 2). Consistent with impaired fractional shortening, animals that had undergone experimental MI had evidence of LV dilatation with increased LV internal diameter in both systole and diastole (LVIDs, LVIDd). Among post-MI rats, LVIDd was significantly reduced by empagliflozin (Table 2, Fig. 1) along with a non-significant numerical reduction in LVIDs (p = 0.11).

**Table 2:** Echocardiographic parameters

	Sham + control	Sham + empa	MI + vehicle	MI + empa
Heart rate (bpm)	382 ± 6	343 ± 6	350 ± 7	336 ± 7
Cardiac output (ml/min)	77 ± 2	65 ± 1*	56 ± 3*^,‡^	50 ± 2*^,‡^
FS (%)	56 ± 1	55 ± 1	21 ± 1*	22 ± 2
LVIDd (mm)	6.8 ± 0.1	6.6 ± 0.1	8.6 ± 0.2*	8.2 ± 0.2*^,†^
LVIDs (mm)	3.0 ± 0.1	3.0 ± 0.1	6.9 ± 0.2*	6.5 ± 0.2

Data are expressed as mean ± SEM. N = 24 in sham + control and sham + empa groups; N = 23 and 25 in MI + vehicle and MI + Empa groups, respectively

*Empa* empagliflozin, *HR* heart rate, *FS* fractional shortening, *LVIDd* left ventricular internal diameter in diastole, *LVIDs* left ventricular internal diameter in systole

* p < 0.05 vs. sham control group

^†^p < 0.05 vs. MI + vehicle group

^‡^p < 0.05 vs. Sham + empa

### Conductance catheterization

Systolic blood pressure (SBP) was measured during cardiac catheterization and was numerically lower in rats that had sustained myocardial infarction consistent with reduced systolic function. It was, however, significantly higher in post-MI rats that had received empagliflozin compared with those that did not (Table 3).

**Table 3:** Invasive pressure volume loop parameters

	Sham + control	Sham + empa	MI + vehicle	MI + empa
SBP (mmHg)	127 ± 4	146 ± 3	120 ± 4*	132 ± 5^†^
dP/dt_max_	7623 ± 326	7903 ± 183	6010 ± 248	6292 ± 249
dP/dt_min_	− 7834 ± 417	− 8234 ± 224	− 4870 ± 251	− 5248 ± 261
PRSW (mmHg)	74 ± 3	80 ± 3	62 ± 5*	75 ± 6^†^
EDP (mmHg)	11.1 ± 0.6	10.2 ± 0.6	16.0 ± 1*	14 ± 1^‡^
Tau (msec)	12.0 ± 0.5	12.1 ± 0.3	17.1 ± 0.5*	16.5 ± 0.5
EDPVR (mmHg/s)	0.029 ± 0.002	0.026 ± 0.002	0.046 ± 0.004*	0.054 ± 0.005

Data are expressed as mean ± SEM. N = 23–24 in sham + control and sham + empa groups; N = 19-23 in MI + vehicle and MI + empa groups

*SBP* systolic blood pressure, *EDP* end-diastolic pressure, *dP/dt*_*max*_ maximum rate of pressure rise, *dP/dt*_*min*_ maximal rate of pressure decline, *EDPVR* end-diastolic pressure volume relationship

* p < 0.05 vs. sham control group

^†^p < 0.05 vs. MI +vehicle group

^‡^p = 0.056 c/w MI + vehicle

#### Invasive load sensitive measures of cardiac function

The maximum pressure developed by the LV during isovolumic contraction, dP/dt_max_, was reduced in post-MI rats and was unaffected by empagliflozin (Table 3). While a frequently used index of systolic function, dP/dt_max_ is influenced by preload, afterload, heart rate, and myocardial mass [6]. The rate at which pressure falls in diastole (− *dP/dt*_*max*_*or* dP/dt_min_) provides a marker of isovolumic relaxation with the caveats that it is affected by alterations in contractility or afterload. In the present study, − *dP/dt*_*max*_ was impaired in post-MI rats but was unchanged by empagliflozin (Table 3).

#### Load insensitive measures of cardiac function

**Fig. 2:**
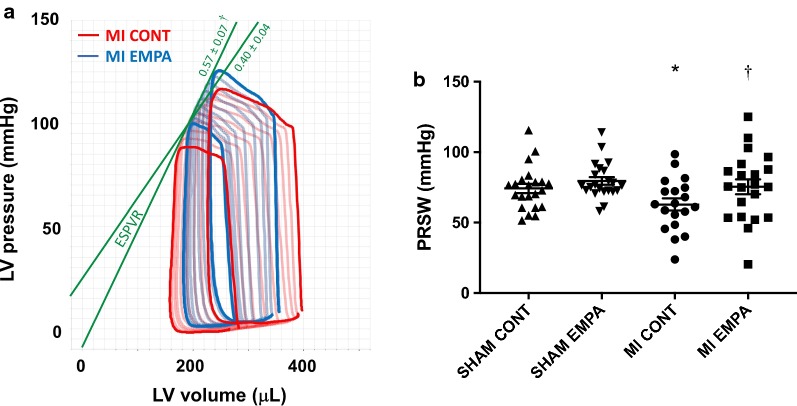
Invasive Pressure Volume loop analysis: while reduced in the post-MI setting, both the end systolic pressure volume relationship, and the preload recruitable stroke work index, load insensitive markers of cardiac contractile function were significantly improved in rats that had received empagliflozin (**a**, **b**). *p < 0.05 MI versus sham, ^†^p < 0.05 versus MI control

When compared with untreated post-MI rats, ESPVR was increased in those animals that had received empagliflozin, indicative of improved cardiac contractility (Fig. 2a). Like ESPVR, the preload recruitable stroke work (PRSW) is insensitive to preload and afterload providing a highly linear index of myocardial contractility. While reduced in the post-MI setting, PRSW was significantly improved in rats that had received empagliflozin (Fig. 2b, Table 3).

Load-independent indices of diastolic function, end-diastolic pressure volume relationship and Tau that reflect the late passive and early active phases of diastole, respectively were impaired in the post-MI setting. Neither was improved with empagliflozin (Table 3).

### Histology

**Fig. 3:**
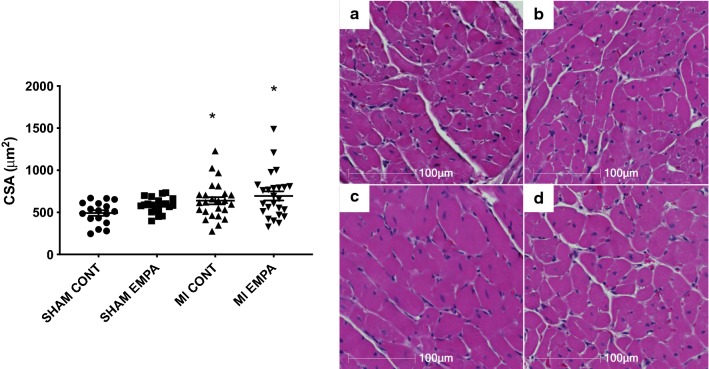
Representative hematoxylin and eosin stained sections. Hearts of MI rats showed evidence of myocyte hypertrophy (Fig. 4c c/w sham animals 4a). Empagliflozin had no effect on either myocyte hypertrophy in either sham or MI animals (Fig. 4b, d) *p < 0.05 MI versus sham

**Fig. 4:**
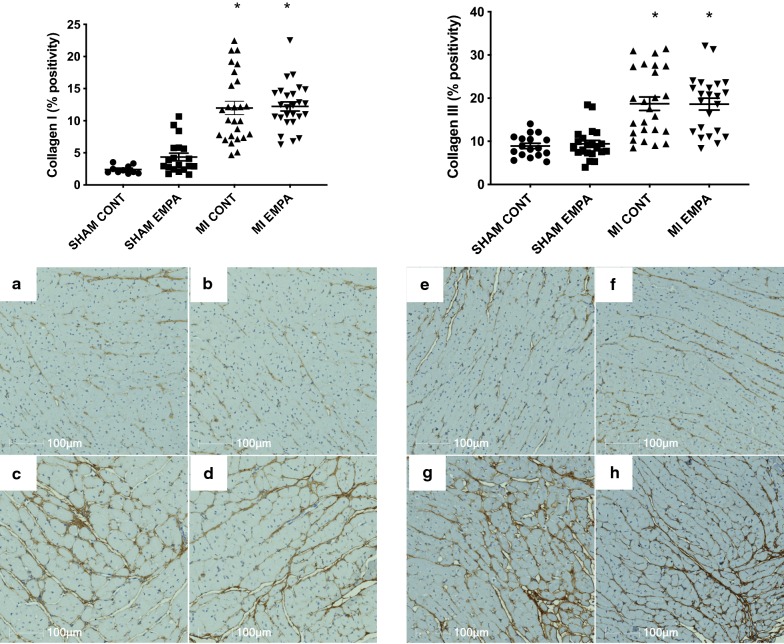
Representative Collagen I and III stained sections. Hearts of MI rats showed evidence of excess extracellular matrix (**c**, G c/w sham animals **a**, **e**). Empagliflozin had no effect on either Collagen I or III positivity in either sham or MI animals (**b**, **d**, **f**, **h**). *p < 0.05 MI versus sham

Cardiomyocyte hypertrophy was assessed by the measurement of cross-sectional area and the extent of fibrosis was quantified by the abundance of collagen types I and III. Myocardial infarction led to remodeling with myocyte hypertrophy (Fig. 3) and increased collagen I, III deposition (Figs. 4a, c, e, g, p < 0.05) that were unaffected by empagliflozin (Figs. 4b, d, f, h).

### Calcium handling proteins

**Fig. 5:**
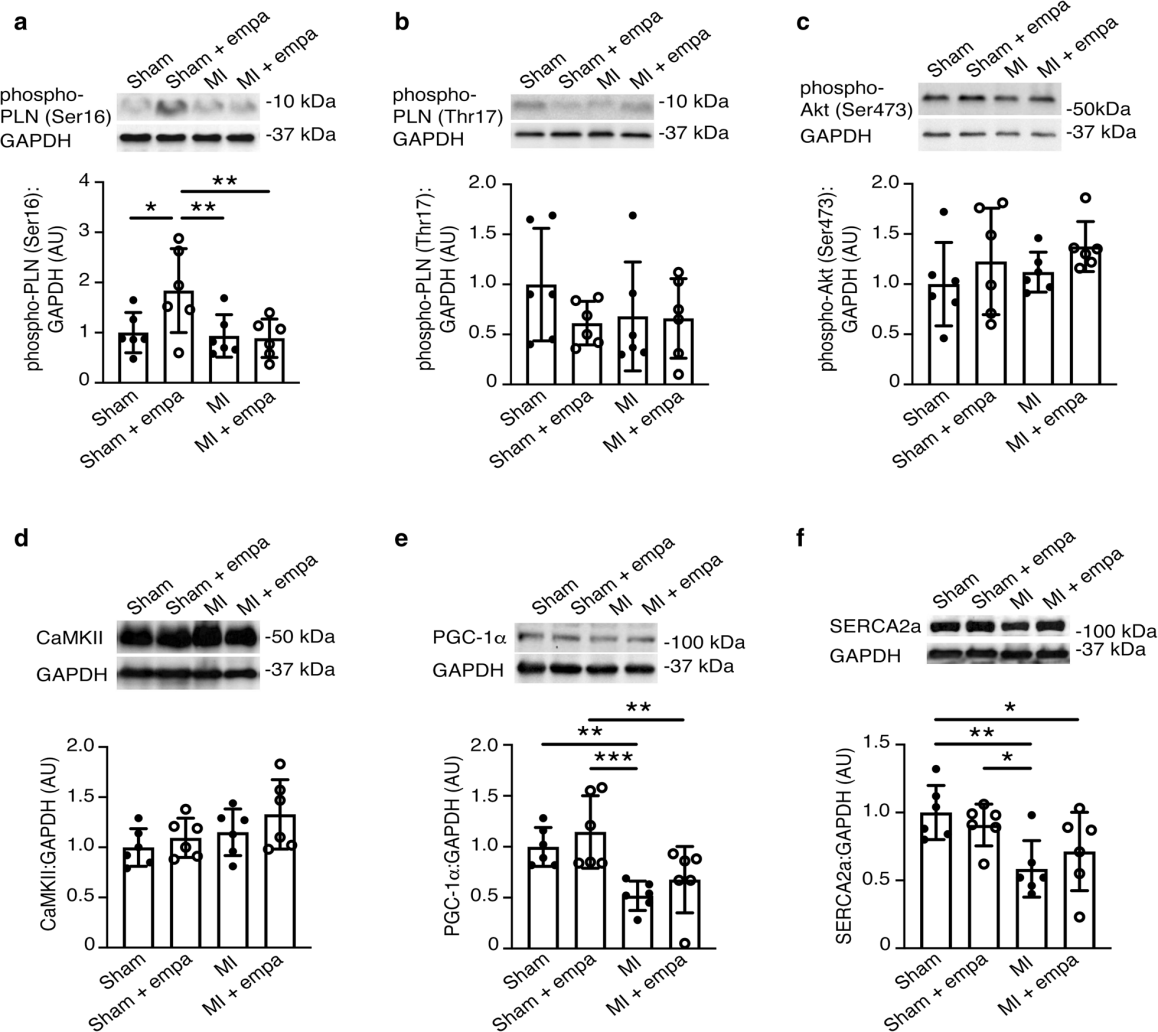
Immunoblotting for cardiac proteins involved in calcium handling and contractility in Fischer F344 rats following sham surgery or left anterior descending artery ligation (MI) and treated with vehicle or empagliflozin (20 mg/kg/day) by oral gavage for 6 weeks beginning 1 week after surgery. **a** Serine 16 phosphorylated phospholamban (PLN). **b** Threonine 17 phosphorylated PLN. **c** Serine 473 phosphorylated Akt. **d** Ca^2+^/calmodulin-dependent protein kinase II (CaMKII). **e** Peroxisome proliferator-activated receptor gamma coactivator-1α (PGC-1α). **f** SERCA2a. *n *= 6/group. Values are mean ± S.D. *p < 0.05, **p < 0.01, ***p < 0.001 by two-way ANOVA followed by Fisher’s least significant difference post hoc test

Given the improvement in cardiac contractility, we assessed protein expression or phosphorylation status of key cardiac proteins involved in calcium handling and contractility (phospho-PLN (Ser16), phospho-PLN (Thr17), phospho-Akt (Ser473), CaMKII, PGC-1α and SERCA2a) (Fig. 5). Myocardial infarction significantly reduced protein expression of SERCA and PGC-1α, whereas it had no effect on phosphorylation of PLN or Akt or on CaMKII protein levels (Fig. 5e, f). In sham-operated rats, empagliflozin treatment significantly increased phosphorylation of PLN on serine residue 16. However, this was not observed in the setting of MI (Fig. 5a). Indeed, there was no difference in phospho-PLN (Ser16), phospho-PLN (Thr17), phospho-Akt (Ser473), CaMKII, PGC-1α and SERCA2a levels between MI rats and MI rats treated with empagliflozin (Fig. 5).

## Discussion

Despite their now well-established ability to reduce heart failure hospitalization in vulnerable patients, the mechanisms whereby SGLT2 inhibitors exert these salutary effects remain elusive. By demonstrating improved systolic function that is independent of loading conditions, the present study provides evidence that this drug class exerts salutary effects on cardiac function beyond those that can be directly ascribed to changes in preload and afterload.

The current study used a post myocardial infarction model, that develops impaired cardiac function and LV dilatation along with structural hallmarks of HFrEF [13]. While dyspnea cannot be directly assessed in rodents, a significant increase in lung weight was noted, consistent with the development of pulmonary congestion. These changes were attenuated by empagliflozin, and notably persisted when lung weight was indexed to tibial length to adjust for the potentially confounding effects of SGLT2 inhibition-induced weight changes.

Because changes in preload and afterload affect the assessment of cardiac function when measured by echocardiography, the interpretation of results obtained with this methodology in the setting of SGLT2 inhibition may be confounded. Conductance cardiac catheterization, on the other hand, can assess cardiac performance independent of loading conditions. Accordingly, we focused on the latter to assess cardiac function in rats with heart failure. Whilst cardiac function was impaired post MI, empagliflozin failed to improve load dependent measures such as fractional shortening consistent with previous reports from our group using dapagliflozin in the post MI setting [12]. In contrast, load independent measures of cardiac contractility were improved in empagliflozin treated animals post MI. We assessed two key load independent parameters, the end-systolic pressure volume relationship (ESPVR) and the preload recruitable stroke work index (PRSW). The ESPVR describes the maximal pressure developed by the LV for any given volume. When systolic function (cardiac contractility, inotropy) becomes impaired, ESPVR becomes flatter and shifts to the right. Conversely, when systolic function improves, ESPVR becomes steeper and shifts to the left. When compared with untreated post-MI rats, ESPVR was increased in those animals that had received empagliflozin, indicative of improved cardiac contractility. Like ESPVR, the preload recruitable stroke work (PRSW) is insensitive to preload and afterload providing a highly linear index of myocardial contractility. Accordingly, when myocardial contractility is reduced so is PRSW and vice versa when contractility is improved. Here, without affecting active or passive relaxation (diastolic function), empagliflozin improved cardiac contractility.

For the past 4 years, following the publication of the first trial that showed a reduction in HHF with SGLT2 inhibition, a plethora of potential mechanisms to account for this effect have been proposed [14] knowing that since SGLT2 is not present in the heart such effects will be secondary. Focussing on cardiac structure as the potential substrate for reduction in heart failure, the present demonstrated a diminution in LV mass and LV end-diastolic diameter with empagliflozin, concordant with cardiac magnetic resonance imaging findings in recently published human studies [15, 16]. These SGLT2 inhibitor-induced changes in LV volume and mass are highly reminiscent of those seen with mineralocorticoid receptor antagonism (MRA) [17], standing in direct contrast to the effects of furosemide where LV end-diastolic volume progressively increases in the presence of LV dysfunction [18]. Together these findings raise the possibility that like an MRA, the initial diuresis induced by SGLT2 inhibition attenuates the development of LV hypertrophy, ultimately leading to improved cardiac function independent of loading conditions.

While an MRA-like effect on volume may account for the improvements observed in the current study, other mechanisms may also contribute. For instance, anaemia, and/or a reduction in hematocrit is a well-delineated adverse prognostic factor in heart failure. MI rats demonstrated a significant increase in hemoglobin. Empagliflozin treatment increased hemoglobin in the sham group, and increased both hemoglobin and plasma sodium in the MI group, further supporting the osmotic diuresis as the most likely candidate for the observed findings. Whether or not this is also driven by increased erythropoiesis was not assessed in the current study, and requires further assessment in human studies.

In keeping with post MI remodeling, we demonstrated myocyte hypertrophy, excessive interstitial fibrosis and a significant reduction in calcium handling proteins such as SERCA2A, and PGC-1α. Intriguingly, none of these structural and molecular parameters were significantly affected by empagliflozin therapy. This is consistent with our prior studies in HFpEF, where key markers of remodeling were not affected by empagliflozin, despite improvements in cardiac function [11]. However, Mustroph et al. studied CaMKII activity and calcium transients in murine and human isolated ventricular myocytes [19]. They demonstrated that empagliflozin reduced CaMKII activity and CaMKII-dependent SR Ca2+ leak, with improvements in Ca2+ transients. The improved Ca2+ transients and calcium mobilization kinetics may in part explain the enhanced contractility seen in the current study. In contrast, Pabel et al. demonstrated that empagliflozin improved diastolic function without altering calcium transient amplitude or diastolic calcium level in human HF isolated cardiomyocytes [20]. Possible explanations for the differences between the current study and the aforementioned studies include the use of isolated myocytes, in contrast to the use of whole heart tissue in the current study, and the differences in techniques used—our study assessed protein expression, in contrast to protein activity. Further, differences in drug concentration used may also explain differences with isolated ventricular myocyte studies using micromolar concentrations, as opposed to the nanomolar concentrations seen in in vivo studies [21].

Our study is not without limitations. Firstly, empagliflozin therapy was commenced 7 days post MI. Further studies are required to assess whether earlier treatment would reverse the structural and functional manifestations of HFrEF. Secondly, although we have postulated a mechanism to account for the beneficial effects that were observed, substantial further investigation will be needed in order to prove that it is, indeed, the case. Thirdly, we did see a reduction in cardiac output in sham + empa animals, in the presence of normal cardiac function, however a small increase in hemoglobin was noted, suggesting this effect was due to osmotic diuresis. Fourthly, we saw a significant increase in systolic blood pressure in MI + EMPA treated animals. In contrast, the DAPA HF study demonstrated a statistically significant reduction in BP of ~ 1.2 mmHg. Whilst we postulate that the increase in BP in the current study was as a result of improved cardiac function and contractility, other parameters such as response to anaesthesia, ventilation etc. may impact vascular tone and resistance, key mediators of systolic blood pressure [22], accounting for the difference’s observed. Fifthly, we did not assess whether empagliflozin therapy impacted MI size or area at risk ex vivo. However, Yoshi et al. [23] demonstrated no impact on SGLT2i therapy upon myocardial injury using an ischemia reperfusion model, and we utilised a well validated echocardiographic technique to assess, and match infarct size prior to randomization [24], thus this is unlikely to account for the observed changes. Finally, we induced MI on young rats, with normal renal function unlike the older populations with comorbidities that account for the vast majority of patients affected by heart failure.

### Clinical implications

The findings of the current study have significant clinical implications. Whereas traditional treatment of HFrEF relies upon modification of neurohumoral activation [1], the current study demonstrated improved contractility, in the absence of typical changes seen with neurohumoral inhibition i.e. no change in myocyte hypertrophy, extracellular matrix or key calcium handling proteins. This is in keeping with the findings of the DAPA HF study, where dapagliflozin improved outcomes in patients with HFrEF, irrespective of glycemic status, and demonstrate only very modest reductions in markers of neurohumoral activation, such as NT-proBNP [25]. Improving contractility without worsening mortality, as occurs with most current inotropes (i.e. milrinone [26]) represents a significant step forward in the management of HFrEF persons—however, the exact mechanism by which this occurs with SGLT2i remains elusive. Further studies, confirming the finding of enhanced contractility in a similar clinical population, along with further studies assessing metabolism etc. are required to better understand this finding.

## Conclusion

In the post myocardial infarction setting, empagliflozin had major beneficial effects on the principal load-independent measures of systolic function, PRSW and ESPVR. Further research is required to assess the mechanisms driving this finding.

## Data Availability

All data is stored on a secure website and available for download, in entirety upon request.

## Abbreviations

HFrEF: Heart failure with reduced ejection fraction
SGLT2i: Sodium–glucose linked cotransporter-2 inhibitor
PRSW: Preload recruitable stroke work
ESPVR: End-systolic pressure volume relationship
LVEDP: Left ventricular end-diastolic pressure
MI: Myocardial infarction
HHF: Hospitalization for heart failure
LVIDs: LV internal diameter in systole
LVIDd: LV internal diameter in diastole
LAD: Left anterior descending coronary artery
